# Evaluation of the Therapeutic Potential of Anti-TLR4-Antibody MTS510 in Experimental Stroke and Significance of Different Routes of Application

**DOI:** 10.1371/journal.pone.0148428

**Published:** 2016-02-05

**Authors:** Lena Andresen, Konstantina Theodorou, Sarah Grünewald, Bozena Czech-Zechmeister, Birte Könnecke, Fred Lühder, George Trendelenburg

**Affiliations:** 1 Department of Neurology, University Medical Center Göttingen, Göttingen, Germany; 2 Department of Neuroimmunology, Institute for Multiple Sclerosis Research, The Hertie Foundation and MPI for Experimental Medicine, Göttingen, Germany; Stanford University, UNITED STATES

## Abstract

Toll-like receptors (TLRs) are central sensors for the inflammatory response in ischemia-reperfusion injury. We therefore investigated whether TLR4 inhibition could be used to treat stroke in a standard model of focal cerebral ischemia. Anti-TLR4/MD2-antibody (mAb clone MTS510) blocked TLR4-induced cell activation *in vitro*, as reported previously. Here, different routes of MTS510 application *in vivo* were used to study the effects on stroke outcome up to 2d after *occlusion of the middle cerebral artery* (MCAO) for 45min in adult male C57Bl/6 wild-type mice. Improved neurological performance, reduced infarct volumes, and reduced brain swelling showed that intravascular application of MTS510 had a protective effect in the model of 45min MCAO. Evaluation of potential long-term adverse effects of anti-TLR4-mAb-treament revealed no significant deleterious effect on infarct volumes nor neurological deficit after 14d of reperfusion in a mild model of stroke (15min MCAO). Interestingly, inhibition of TLR4 resulted in an altered adaptive immune response at 48 hours after reperfusion. We conclude that blocking TLR4 by the use of specific mAb is a promising strategy for stroke therapy. However, long-term studies with increased functional sensitivity, larger sampling sizes and use of other species are required before a clinical use could be envisaged.

## Introduction

Ischemic brain damage after stroke results from a complex pattern of pathophysiological events including excitotoxicity, periinfarct depolarizations, inflammation and programmed cell death [1]. The important contribution of immune-mediated mechanisms, including the activation of innate immune receptors such as Toll-like receptors (TLRs), has been increasingly recognized over the last decade [2,3]. TLRs represent a family of transmembrane pattern-recognition receptors, which during infections recognize various conserved structural motifs, named pathogen-associated molecular patterns (PAMPs). However, TLRs can also be activated by endogenous ‘danger signals’ called DAMPs (danger-associated molecular patterns), which are released from injured or stressed cells under situations of sterile inflammation or ischemia [3]. There are several reports showing that TLRs mediate ischemic brain injury and TLR2 deficient mice were protected against ischemic stroke [4,5,6]. Intravascular applied monoclonal antibodies permeate rodent brain after induction of focal cerebral ischemia [7]. Specifically, the application of TLR2 blocking T2.5 antibody *in vivo* demonstrated the anti-inflammatory effect of TLR2-inhibition in experimental stroke [8]. However, TLR2 inhibition can cause complications such as a hampered neuroplasticity or dysregulated immune responses, as reported recently by Bohacek et al. [9].

Besides TLR2, TLR4 is also highly induced after cerebral ischemia [6], TLR4 deficient mice were protected against ischemic stroke [5,10,11,12], and polymorphisms of the TLR4 gene were found to be associated with stroke occurrence in a Chinese population [13]. Moreover, a recent study revealed that intracerebroventricular injection of the pharmacological TLR4-NOX4 signal inhibitor resatorvid protects against neuronal death in transient focal ischemia [14].

Therefore, we investigated if and by which route (*i*.*a*., *i*.*v*., *i*.*p*.) pharmacological *in vivo*-TLR4-inhibition after ischemic stroke would protect against ischemic brain injury without the detrimental long-term effects observed in TLR2-deficient mice [9]. For this purpose we determined the infarct volume, inflammatory cell accumulation and neurological deficit after TLR4 inhibition after MCAO.

Our results demonstrate that intravascular (*i*.*v*. or *i*.*a*.) TLR4 application robustly protects against ischemic injury for at least 48h after reperfusion. We did not observe any long-term detrimental effect of TLR4 inhibition. Interestingly, TLR4 inhibition not only alters the innate immune response, but also changes T-cell related adaptive immune response.

## Materials and Methods

### Animals

8–10 weeks old, male C57Bl/6N wild-type mice (Charles River, Cologne, Germany; Janvier Labs S.A.S., St Berthevin, France) were housed under diurnal lighting conditions and allowed access to food and water *ad libitum*. All animal handling and surgery were performed according to the Guidelines for the Use of Animals in Neuroscience Research (Society for Neuroscience) and to institutional and national guidelines. All experiments were approved by the local institutional Animal Care Committee, LaVeS (No.33.9-42502-04-12/849). All experiments were performed in a randomized manner, and by investigators blinded to the groups of treatment.

### TLR4 inhibition *in vitro*

Murine macrophage RAW 264.7 cells were grown in high glucose DMEM (Biochrom KG, Berlin, Germany) supplemented with 10% FCS (Biochrom KG, Berlin, Germany). Cells were preincubated with or without 50μg/ml rat anti-mouse anti-TLR4/MD-2 monoclonal antibody MTS510 (eBioscience Inc., San Diego, CA) for 30min and subsequently stimulated with 10ng/ml LPS (Sigma-Aldrich, Munich, Germany) as a TLR4 agonist. Six hours after stimulation, supernatants were used for a TNF bioassay, which was performed in triplicates as described previously [8].

### MCAO and therapeutic TLR4 inhibition *in vivo*

1 μg (*i*.*a*. injection) or 2 μg (*i*.*v*. or *i*.*p*. injection) of MTS510 (eBioscience Inc., San Diego, CA) was applied to inhibit TLR4 signaling *in vivo* [15]. 1 μg (*i*.*a*. injection) or 2 μg (*i*.*p*. injection) Isotype rat IgG2a K (eBioscience, San Diego, USA) or PBS was used as control. For *i*.*v*. and *i*.*p*. treatment MTS510 was applied at reperfusion time points 0 h and 24 h (2 x 1μg/animal). The antibody was applied only once because of technical limitations in experiments with *i*.*a*. injection of MTS510, whereas it was applied twice in *i*.*p*. and *i*.*v*.-experiments. We speculated that a higher concentration of anti-TLR4 antibody in the animals inhibits TLR-mediated pro-inflammatory signaling more efficiently, as demonstrated recently by others *in vitro* and *in vivo* [16,17,18]. Middle cerebral artery occlusion (MCAO) was performed as described previously [19,20]. Mice were anaesthetized with 5% isoflurane in 100% oxygen with a flow of 0.8 l/min and maintained anaesthetized during MCAO procedure with 1% isoflurane. They were kept under spontaneous respiration. Before and directly after suturation ointment containing dexpanthenole was placed onto the animals eyes to prevent dehydration. Analgetic treatment included intraperitonally applied buprenorphine (0.1 mg/kg body weight) during surgery and lidocaine gel placed onto the sutures directly after suturation as well as 24 hours after MCAO. The animal cages were kept on heating pads to maintain a constant cage temperature of 24°C until 72h after reperfusion (see also S1 Text).

### Exclusion and euthanasia criteria

Animals that died within 6 hours after MCAO were excluded from any analysis as death was assumed to be a direct complication of the surgical procedure. To ensure human endpoints during the study, specific euthanasia criteria were defined (see also local ethic approval LaVeS / No.33.9-42502-04-12/849) according to which animals that had lost 20% of their initial body weight within 48 hours or had been measured surficial body temperatures lower than 24°C without recovery within 24 hours were deeply anaesthetised, then cervically dislocated and finally decapitated. Even though body weight and surficial body temperature were only documented and analysed before MCAO and 24, 48 and 72 hours as well as 7 and 14 days after reperfusion, the animals were daily seen for health monitoring (S1 Text).

### Neurological Scoring

Neurological deficits were assessed before, 24h and 48h after a 45min MCAO, and 2h, 7d, and 14d after a 15min MCAO. Neurological sensomotor deficits were graded as described by Bederson [21] and modified by Hara [22]: 0—no deficit, 1—failure to extend left paw, 2—circling to the left, 3—no spontaneous activity, and 4—death of the animal. Mice that died within 6h after the MCAO procedure were excluded from the experiments.

### Mortality rates

MTS510 *i*.*a*.: no animal died during or within the first 6h after MCAO. MTS510 *i*.*p*. after 45min MCAO: 2 out of 30 mice were excluded because of perioperative death during MCAO (during, or up to 6h after MCAO); MTS510 *i*.*v*. after 45min MCAO: 2 out of 30 mice were excluded because of perioperative death; MTS510 *i*.*v*. after 15min MCAO: 2 out of 40 mice were excluded because of perioperative death, and 3 due to insufficient occlusion of the *middle cerebral artery* as measured by laser doppler flowmetry.

### Determination of lesion sizes

48h or 14d after reperfusion animals were deeply anaesthetized and brains were removed from the skull. Brain tissue was cut into slices of 2 mm depth. In order to measure the size of the ischemic lesion 48h after start of reperfusion, 2,3,5–triphenyl-tetrazolium-chloride (TTC) staining was used. Both sides of each section were scanned and infarct volumes were measured with the National Institutes of Health Image J software (NIH, USA). The size of the ischemic lesion 14d after reperfusion was measured with FlouroJade C (S1 Text). Brain swelling/edema was calculated by subtracting the size of the whole contralateral (non-infarcted) hemisphere from the whole ipsilateral (infarcted) hemisphere, and represents the difference between direct and indirect infarct volumes [20].

### Immunofluoresence of brain tissue

Mice were euthanized at 48h, or 14d, respectively, after induction of MCAO. Brains were removed after transcardial perfusion, post-fixed in 4% PFA and 30% sucrose (each over night) and embedded for cryogenic cuttings. 12μm sections were air-dried and blocked at 4°C over night using a solution of 0,25% Triton-X-100 and 5% donkey serum in TBS followed by an incubation with mouse monoclonal NeuN-antibody (1:100–1:500, Merck Millipore, USA), rabbit polyclonal Iba1-antibody (1:500–1:1000, Wako Chemicals GmbH, Neuss) and chicken polyclonal GFAP-antibody (1:1000, Merck Millipore, USA) for 24h at 4°C. Incubation with secondary antibodies (Cy5-conjugated donkey anti-mouse, Cy3-conjugated donkey anti-rabbit, and FITC-conjugated donkey anti-chicken) was performed for 60min at room temperature in the dark, and followed by staining with DAPI. Slides were scanned with AxioCamMRm and processed with Zen software Version 1.0.0.0 (Zeiss, Jena, Germany). Cells were counted using Image J software (NIH, USA).

### Flow cytometry

For FACS analysis, whole brain hemispheres were separated and stored in PBS on ice for one hour. Brain-derived cells were isolated using 30%, 45%, and 75% of Percoll gradients (BD-Bioscience, San Jose, CA, USA). In order to specify the cell populations the following antibody markers were used: CD3-biotin (1:200, clone 15-2C11, BD Bioscience), CD11b-APC (1:300, clone 111/70, Biolegend, San Diego, CA), CD45.2-FITC (1:100, clone 104, Biolegend), CD45 B220-PE (1:300, clone RA3-6b2, BD Bioscience), and APC/Cy7 (Biolegend). Antibodies were incubated for 15min at a temperature of 4°C in the dark. Cells were analyzed using FACS Aria (BD Bioscience) and FACSDiva Version 6.1 software. Specific cell numbers were normalized to 200.000 cells analyzed.

### Statistical analysis

Power calculation was performed using SISA-Binominal as described before [23]. Based on the known variance of previous experiments the MCAO experiments were powered (*α* = 0.05; *β* = 0.8) to detect effect sizes *d* (ref. [24]) of at least 1, i.e. of one standard deviation. For comparison of infarct volumes, neurological deficit, or cell numbers one-tailed Mann-Whitney-U-test was used (GraphPadPrism Software Version 5, La Jolla, USA) if not stated otherwise. Evaluation of longterm outcome was based on two-tailed analyses. p-values below 0.05 were considered statistically significant. Data are presented as single values (*scatter blots*) combined with the mean ± SD, if not stated otherwise. Neuroscore graphs show single values and the median.

## Results

### Anti-TLR4 antibody blocks TLR4-mediated signaling *in vitro*

**Fig 1:**
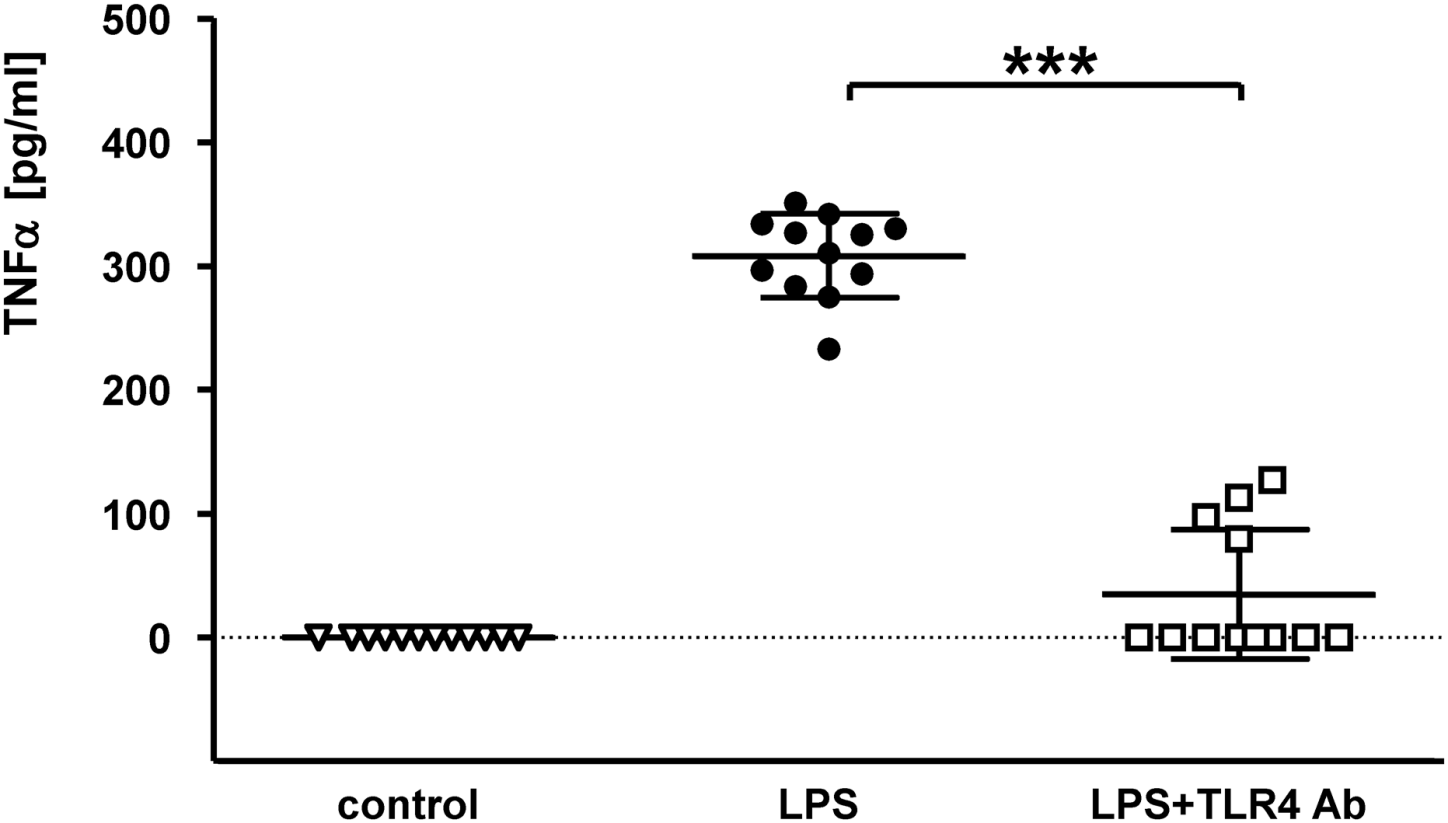
Anti-mouse TLR4/MD2 antibody clone MTS510 suppresses TLR4-mediated immune response *in vitro*. Murine RAW264.7 macrophage cells were incubated with (or without) anti-TLR4/MD2 (clone MTS510) antibody for 30min before incubation of the cells for 2 days with 10ng/ml lipopolysaccharide (LPS). TNFα concentration in the supernatant was determined by the use of a bioassay with the L929 reporter cell line and supernatant (1:200 dilution). TNFα concentration is indicated in pg/ml and was determined with 4 measurements of three separate biological samples in each group (**control**: incubation without LPS and MTS510 antibody; **LPS**: incubation with LPS only; **LPS+TLR4**: incubation with MTS510 antibody before stimulation with LPS), data are present with the mean ± SD (***p< 0.0001 as determined by Mann-Whitney U test).

To evaluate if the monoclonal anti-TLR4 antibody clone MTS510 (ref. [25]) blocks TLR4 activation *in vitro*, murine macrophage RAW 264.7 cells were stimulated with the TLR4-agonist LPS (10ng/ml) with or without previous incubation with MTS510. The tumor necrosis factor-α concentration was significantly reduced in the supernatant of cells incubated with the anti-TLR4 antibody after stimulation with LPS compared with untreated cells (mean TNFα concentration [pg/ml] ± SD: TNFα_*(LPS)*_: 308.2l ± 33.7 pg/m; TNFα_*(LPS+TLR4mAb)*_: 34.7 ± 52.4 pg/ml; p < 0.0001) (Fig 1). Thus, MTS510 anti-TLR4 antibody efficiently inhibits TLR4-mediated proinflammatory signaling *in vitro*.

### Evaluation of the therapeutic potential of anti-TLR4 antibody *in vivo* applied intraperitoneally (*i*.*p*.)

**Fig 2:**
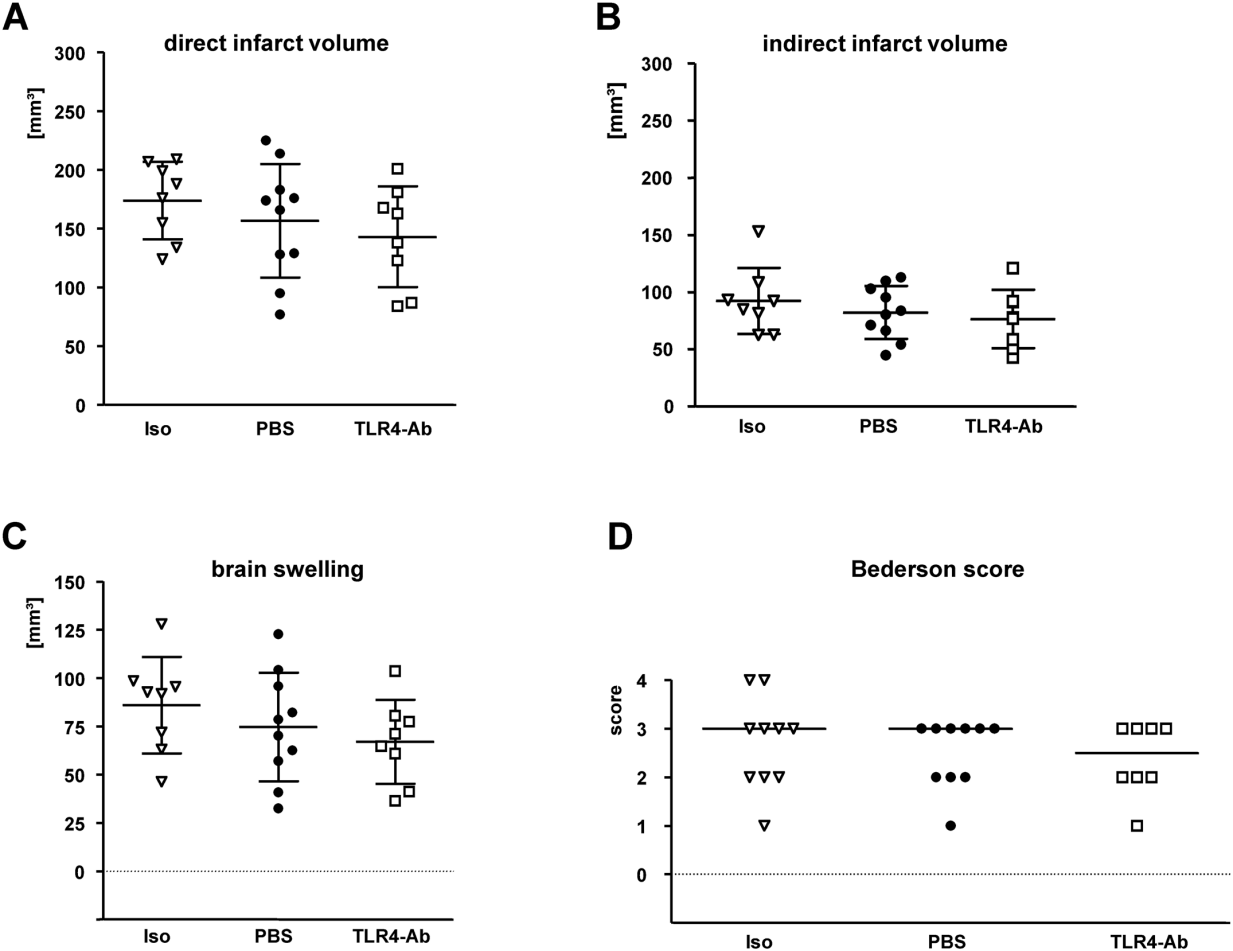
Evaluation of the therapeutic potential of of anti-TLR4 mAb *in vivo* applied *i*.*p*.. MTS510 mAb (**TLR4-Ab**), or isotype control antibody (**Iso**) were applied *i*.*p*. at the end of 45min MCAO and once again after 24h of reperfusion in a dose of 1μg/animal in each application. A second control was performed by omission of any antibody and thus pure injection of vehicle (**PBS**). A correction for edema (brain swelling) was applied by calculating the indirect infarct volume as the volume of the contralateral hemisphere minus the non-infarcted volume of the ipsilateral hemisphere. The difference between direct (**A**) and indirect (**B**) infarct volumes represents brain swelling (**C**). Neurological deficit (**D**) was ranked according to a modified Bederson-score from 0 (no deficit) to 4 (dead). Data are presented as single values (*scatter blots*) combined with the mean ± SD; neuroscore graph shows single values and median (n_*Iso*_ = 10; n_*PBS*_ = 10; n_*TLR4Ab*_ = 8).

To examine if the application of anti-TLR4/MD2 mAb protects against ischemic injury *in vivo*, 1μg MTS510 was applied intraperitoneally (*i*.*p*.) at the start of reperfusion and at 24h of reperfusion after 45min MCAO. Because of the adverse effects of unspecific antibodies in a recent study [8], PBS and an isotype control antibody were used as separate control treatments. As shown in Fig 2, there was a tendency towards reduced infarct volumes in the anti-TLR4 treated group when compared to the two control groups, however without statistical significance (direct infarct volumes_[mean±SD]_: isotype control 174.0 ± 33.0 mm^3^, PBS 156.7 ± 48.5 mm^3^, TLR4mAb 143.1 ± 42.9 mm^3^; indirect infarct volumes_[mean±SD]_: isotype control 92.4 ± 28.9 mm^3^, PBS 82.3 ± 23.3 mm^3^, TLR4mAb 76.5 ± 25.5 mm^3^; brain swelling_[mean±SD]_: isotype control 86.0 ± 25.0 mm^3^, PBS 74.6 ± 28.1 mm^3^, TLR4mAb 67.0 ± 21.7 mm^3^; p > 0.05) (Fig 2A–2C). The neurological deficit at 48h after reperfusion also showed only a tendency without significance in TLR4mAb treated wild-type mice when compared to PBS or isotype control (median[25% percentile/75% percentile]: isotype control 3[2/3,25], PBS 3[2/3], TLR4mAb 2.5[2/3], p > 0.05) (Fig 2D). Moreover, evaluation of absolute cell numbers of neurons and inflammatory (Iba1-positive) cells in TLR4-mAb treated or vehicle-treated control animals revealed no significant difference between the two treatment groups at 48h after MCAO (NeuN positive cell count in the ischemic hemisphere: PBS 71.3 ± 20.2 / mm^2^, TLR4mAb 69.3 ± 17.5 / mm^2^; activated Iba1-positive cell count in the ischemic hemisphere: PBS 15.5 ± 4.8 / mm^2^, 15.8 ± 5.2 / mm^2^; p > 0.05) (S1 Fig).

### Evaluation of the therapeutic potential of anti-TLR4 antibody *in vivo* applied intra-arterially (*i*.*a*.)

**Fig 3:**
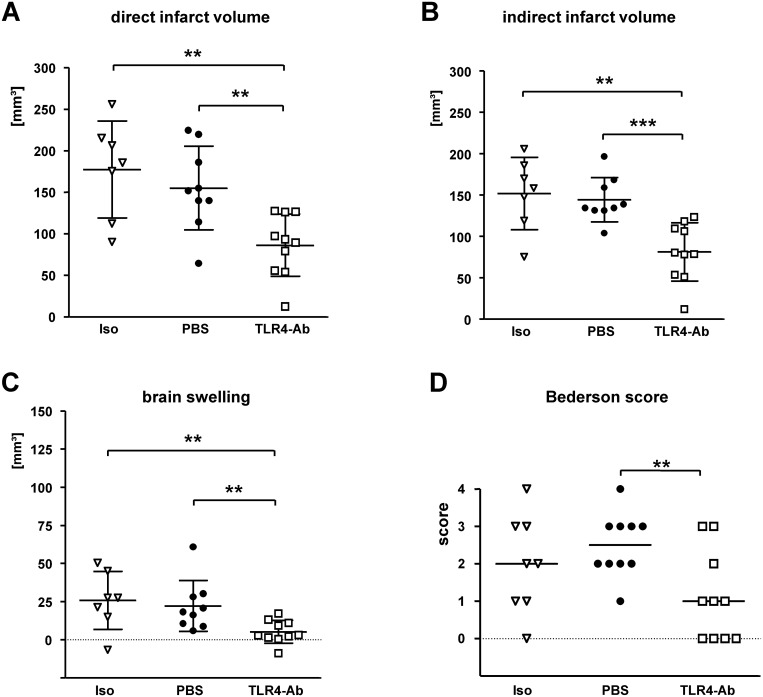
Evaluation of the therapeutic potential of anti-TLR4 mAb *in vivo* applied *i*.*a*.. MTS510 mAb (**TLR4-Ab**), isotype control (**Iso**), or vehicle (**PBS**) was applied *i*.*a*. at the start of MCAO (45min) in a dose of 1μg/animal in each application. Besides application of isotype control antibody, a second control was performed by omission of any antibody and injection of vehicle only (**PBS**). The difference between direct (**A**) and indirect (**B**) infarct volumes at 48h of reperfusion represents brain swelling/edema (**C**). Neurological deficit (**D**) at 48h of reperfusion after 45min of MCAO was ranked according to a modified Bederson-score from 0 (no deficit) up to 4 (dead). Data are presented as single values (*scatter blots*) combined with the mean ± SD; neuroscore graph shows single values and the median (***p < 0.001; **p < 0.01; *p < 0.05; Mann-Whitney U test; number of animals in treatment groups: n_*iso*_ = 8, n_*PBS*_ = 10, n_*TLR4Ab*_ = 10).

MTS510 was applied *i*.*a*. to test our hypothesis that lack of significant protection in mice treated with MTS510 *i*.*p*. could be due to insufficient transport of MTS510 to the ischemic brain tissue, potentially resulting in only low antibody-concentrations in the ischemic brain tissue. Indeed, mice treated with 1μg anti-TLR4/MD2 mAb MTS510 *i*.*a*. at the beginning of induction of MCAO had highly significant smaller infarct volumes at 48h after reperfusion than mice treated with vehicle (PBS) only or isotype control antibody (direct infarct volumes_[mean±SD]_: isotype control 177.4 ± 58.3 mm^3^, PBS 155.2 ± 50.4 mm^3^, TLR4mAb 86.3 ± 37.4 mm^3^; p_*[PBS vs TLR4mAb]*_ 0.0015, p_*[iso vs TLR4mAb]*_ 0.0034) (Fig 3A). The same was true in regard to indirect infarct volumes (mean±SD: isotype control 151.8 ± 43.6 mm^3^, PBS 144.1 ± 26.7 mm^3^, TLR4mAb 81.1 ± 35.1 mm^3^; p_*[PBS vs TLR4mAb]*_ 0.0001; p_*[iso vs TLR4mAb]*_ 0.0034) (Fig 3B) or brain swelling/edema (mean±SD: isotype control 25.7 ± 19.0 mm^3^, PBS 22.2 ± 16.7 mm^3^, TLR4mAb 5.17 ± 7.5 mm^3^; p_*[PBS vs TLR4mAb]*_ 0.0028; p_*[iso vs TLR4mAb]*_ 0.0068) (Fig 3C). Evaluation of the neurological deficit at 48h after MCAO in *i*.*a*.-treated animals also revealed a highly significant improvement in MTS510-treated animals when compared to PBS-treated control mice (Bederson score, median [25%percentile/75%percentile]: isotype control 2 [1/3], PBS 2.5 [2/3], TLR4mAb 1 [0/2.25]; p_*[PBS vs TLR4mAb]*_ 0.0051) (Fig 3D).

### Evaluation of the therapeutic potential of intravenous (*i*.*v*.) application of anti-TLR4 antibody *in vivo*

Next, we wondered if systemic intravascular (*i*.*v*.) application of MTS510 at the end of MCAO could also protect against experimental stroke, since this would fit better with a potential clinical application in stroke patients. Only PBS-treatment was used in control mice for the evaluation of the therapeutic potential of *i*.*v*. application of anti-TLR4 antibody *in vivo*, because of similar results in both control groups (PBS vs. isotype control antibody treated mice) when animals were treated *i*.*a*. or *i*.*p*. (see above).

**Fig 4:**
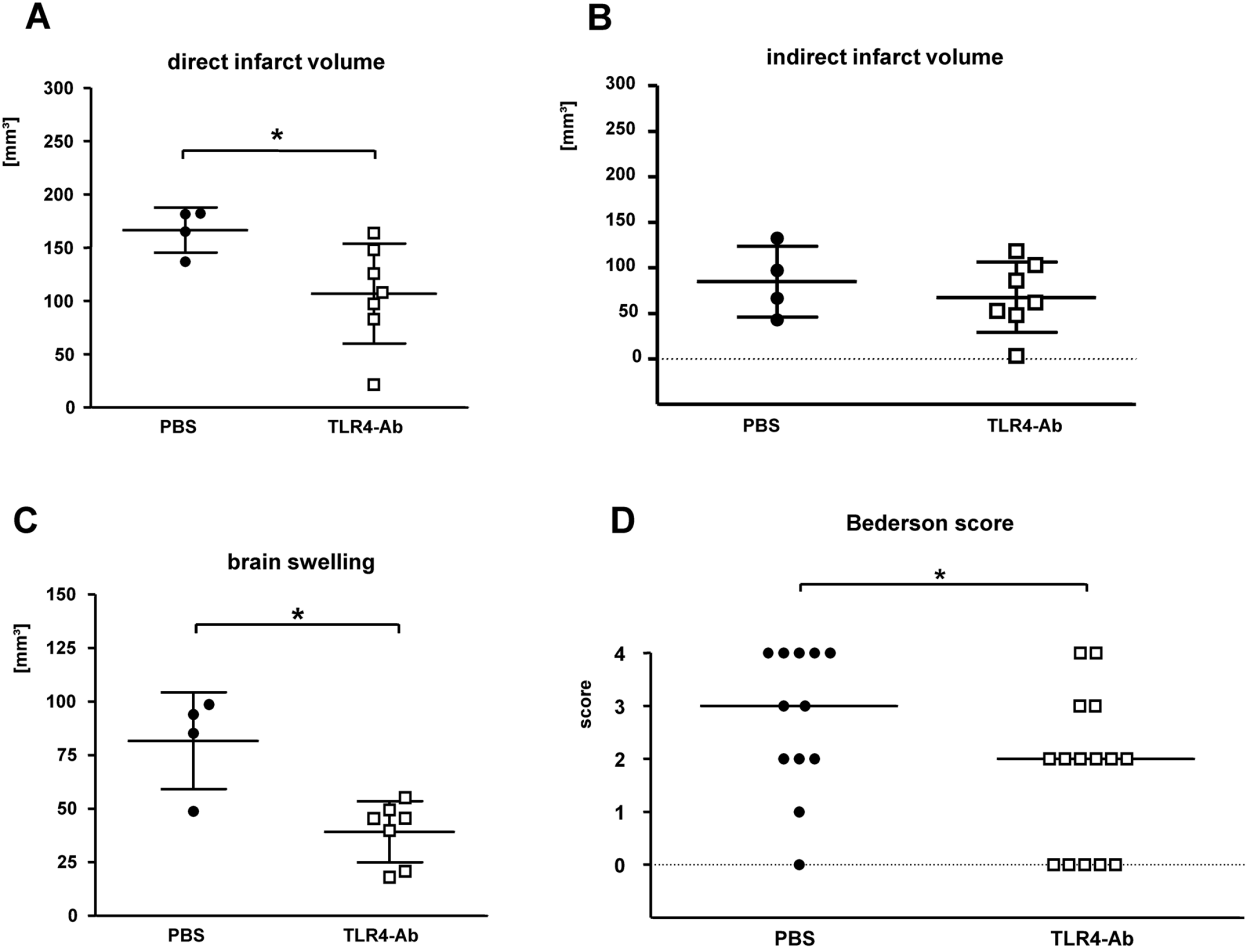
Evaluation of the therapeutic potential of anti-TLR4 mAb *in vivo* applied *i*.*v*.. MTS510 mAb (**TLR4-Ab**), respectively vehicle (**PBS**) was applied *i*.*v*. at the end of MCAO for 45min and once again after 24h of reperfusion in a dose of 1μg/animal each application. A second control was performed by omission of any antibody and pure injection of vehicle (**PBS**). The difference between direct (**A**) and indirect (**B**) infarct volumes represents brain swelling/edema (**C**). Neurological deficit (**D**) at 48 h of reperfusion was ranked according to a modified Bederson-score from 0 (no deficit) up to 4 (dead). Data are presented as single values (*scatter blots*) combined with the mean ± SD; neuroscore graph shows single values and the median (*p< 0.05; Mann-Whitney U) (n_*PBS*_ = 12; n_*TLR4Ab*_ = 15).

Indeed, mice treated with anti-TLR4/MD2 mAb MTS510 *i*.*v*. twice (at start of reperfusion and after 24h) had significant smaller direct infarct volumes (mean±SD: PBS 166.4 ± 21.2 mm^3^, TLR4mAb 106.7 ± 47.1 mm^3^; p_*[PBS vs TLR4mAb]*_ 0.012) (Fig 4A). This effect was also observed with regard to brain swelling/edema (mean±SD: PBS 81.7 ± 22.6 mm^3^, TLR4mAb 39.3 ± 14.3 mm^3^; p_*[PBS vs TLR4mAb]*_ 0.012) (Fig 4C), but not the indirect infarct volume (mean±SD: PBS 84.8 ± 38.8 mm^3^, TLR4mAb 67.5 ± 38.6 mm^3^; p_*[PBS vs TLR4mAb]*_ > 0.05) (Fig 4B). Evaluation of the neurological deficit at 48h after MCAO in *i*.*v*.-treated animals also revealed a better performance of MTS510-treated mice when compared to PBS-treated controls (Bederson score, median[25%percentile/75%percentile]: PBS 3[2/4], TLR4mAb 2[0/3]; p_*[PBS vs TLR4mAb]*_ 0.0385) (Fig 4D). Thus, *i*.*v*. MTS510 treatment protected against ischemic injury, however not with the significance level observed after *i*.*a*. treatment.

### Evaluation of the long-term effects of anti-TLR4 treatment after stroke

Due to a recent report on the detrimental effects of TLR2-deficiency after experimental ischemia in the long-term [9], we evaluated if the potential detrimental effects of TLR4-inhibiton occur up to 14d after induction of cerebral ischemia. There was no difference with regard to physiological parameters, such as temperature or mean body weight between the treatment groups (temperature [mean±SD]: PBS 30.4 ± 2.3°C, TLR4mAb 30.2 ± 2.3°C; body weight [mean±SD]: PBS 22.6 ± 2.1 g, TLRmAb 22.0 ± 2.0 g; p > 0.05) (S2 Fig). 15min of MCAO results in a relevant infarcted area at 14d after reperfusion (S3 Fig). At 14d after reperfusion, there was no significant alteration of infarct sizes in mice treated with anti-TLR4mAb (1μg MTS510 *i*.*a*.) when compared to control mice (infarct size [mean±SD]: PBS 5.7 ± 3.8 mm^3^, TLR4mAb 5.1 ± 4.2 mm^3^; p > 0.05) (S4A Fig) despite a tendency towards a better neuronal survival in anti-TLR4-treated animals, however without statistical significance (ratio of NeuN positive cells_ipsilateral/contralateral hemisphere_ [mean ± SD]: PBS 0.79 ± 0.12, TLR4mAb 0.84 ± 0.12; p > 0.05) (S4B Fig). A similar situation was observed when ipsilateral astrocytes or activated microglia/macrophages were evaluated, there being no significant difference between the treatment groups (GFAP positive cells [mean ± SD]: PBS 120.0 ± 37.6 mm^2^, TLR4mAb 130.0 ± 61.7 mm^2^; activated Iba1-positive cells [mean ± SD]: PBS 54.4 ± 44.6 mm^2^, TLR4mAb 61.4 ± 48.2 mm^2^; p > 0.05) (S4C and S4D Fig). In accordance to this, no significant differences in neurological deficits were detected by the Bederson score nor the mNSS (modified Neurological Score Scale) (animals after 14d [median]: mNSS_14dPBS_ 15; mNSS_14dTLR4mAb_ 15; p > 0.05). Thus, this data rather argues against a potential detrimental long-term effect of TLR4-inhibition. Accordingly, neither did a Kaplan-Meier analysis of animal survival show any significant difference between the two treatment groups, but rather a (non-significant) tendency towards a better survival in anti-TLR4-treated mice (median survival time PBS: 96d, TLR4mAb: 121d; p > 0.05) (S5 Fig).

### Analysis of inflammatory cell types after anti-TLR4 treatment *i*.*v*. by flow cytometry

**Fig 5:**
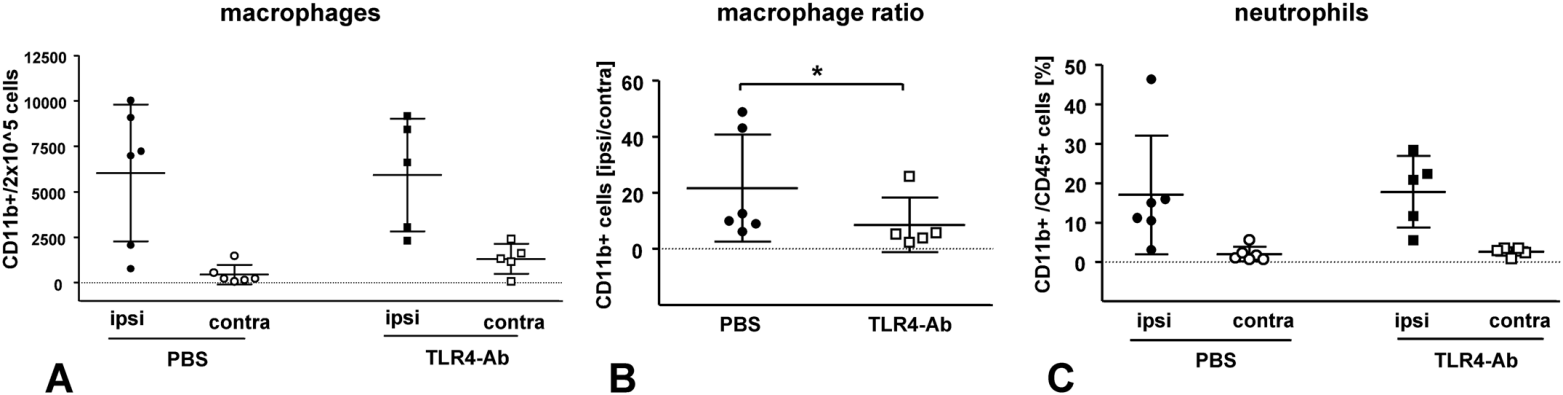
Evaluation of inflammatory cell counts in whole hemispheres of *i*.*v*. vehicle, and anti-TLR4mAb treated wild-type mice at 48h after 45min MCAO. Total cell count in each hemisphere was determined by the use of flow cytometry. CD11b-positive cells (activated macrophages/microglia) were shown with their absolute cell counts (**A**) in each, ispilateral/ischemic (**ipsi**) and contralateral/non-ischemic (**contra**) hemisphere of PBS- or anti-TLR4mAb-treated mice; as well as with the ratio of induction in ischemic hemisphere when compared to contralateral/non-ischemic hemisphere (**B**). Fig 5C shows the number of neutrophils identified in whole ischemic and non-ischemic hemispheres of PBS and anti-TLR4-mAb treated mice (mean values ± SD; *p< 0.05; Mann-Whitney U; n_*PBS*_ = 6; n_*TLR4Ab*_ = 5).

Whether or not TLR4-inhibition affects the post-ischemic immune response was evaluated by the use of flow cytometry (FACS). Complete hemispheres of vehicle- or anti-TLR4 mAb-*i*.*v*.-treated wild-type mice at 48h after 45min MCAO were collected and analyzed with regard to the total cell number of different immune cell types in the whole ipsilateral/ischemic and contralateral/non-ischemic hemisphere (Fig 5). Absolute numbers of activated microglia/macrophages (Fig 5A) were not altered at 2d after 45min MCAO in MTS510-treated mice when compared to vehicle-treated control mice; neither in the ischemic, nor in the non-ischemic hemisphere (CD11b positive cells [mean ± SD / 2 x 10^5^]: PBS_ipsi_ 6034 ± 3767 / 2 x 10^5^ cells, TLR4mAb_ipsi_ 5927 ± 3099; p > 0.05). However, anti-TLR4-treatment resulted in a small increase of the number of activated macrophages/microglia in the contralateral/non-ischemic hemisphere, which led to a decrease of the macrophage/microglia-ratio between both hemispheres (ratio of CD11b positive cells in the ispilateral/contralateral hemisphere [mean ± SD]: PBS_CD11b ratio ispi/contra_ 21.6 ± 19.1, TLR4mAb_CD11b ratio ispi/contra_ 8.6 ± 9.8; p = 0.026) (Fig 5B). Neutrophil counts were not altered after MTS510-treatment when compared to control treatment (percentage of CD11b^+^CD45^+^ cells [mean ± SD]: PBS_ipsi_ 17.0 ± 15.1, TLR4mAb_ipsi_ 17.8 ± 9.1; p > 0.05), as shown in (Fig 5C).

### Analysis of cells of the adaptive immune system after anti-TLR4 treatment *i*.*v*. by flow cytometry

**Fig 6:**
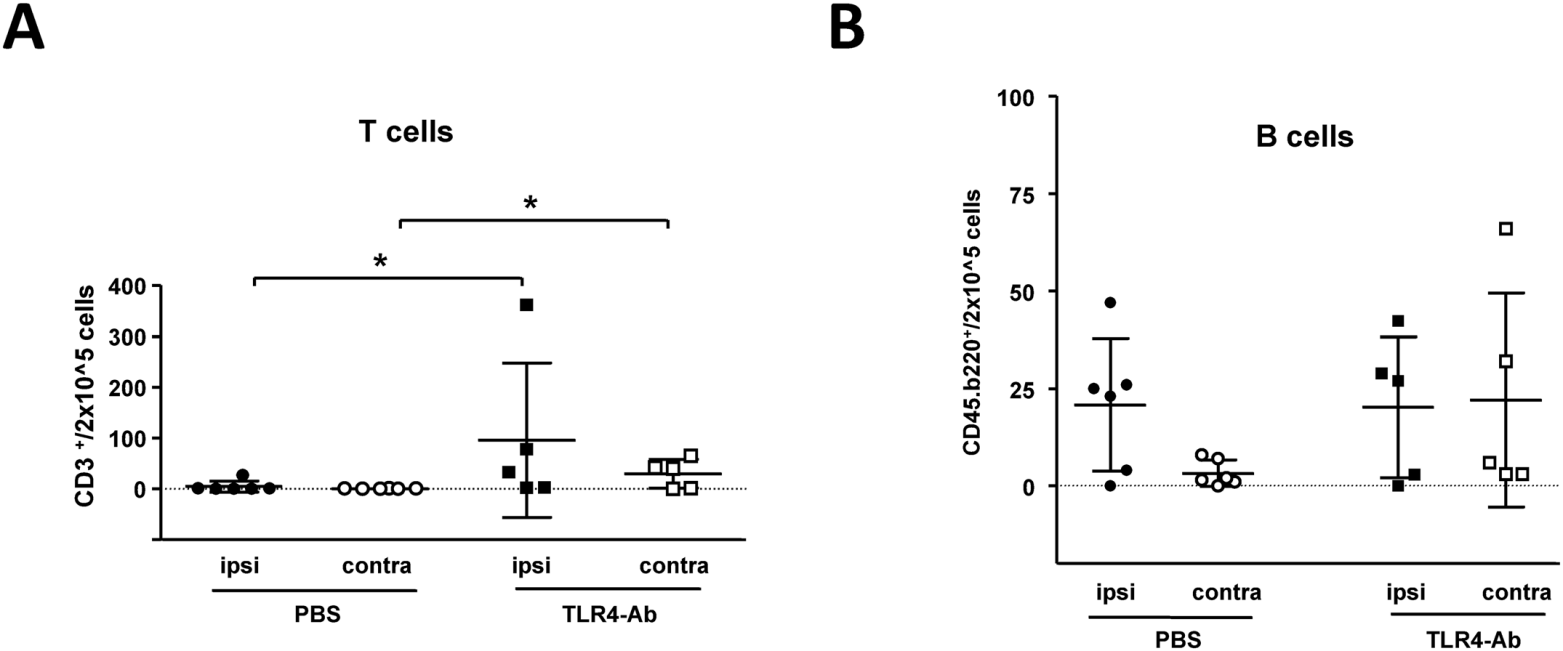
Evaluation of cells of the adaptive immune response at 48h after 45min MCAO in mice *i*.*v*. treated with anti-TLR4mAb or vehicle control. Through CD3- or CD45.B220-specific selection by the use of flow cytometry, the total T-cell count (**A**), and the total B-cell count (**B**) in whole ischemic (**ipsi**), and non-ischemic (**contra**) hemispheres of *i*.*v*. TLR4mAb treated (**TLR4-Ab**) and vehicle (**PBS**) treated mice was determined at 48h after 45min of MCAO (mean values ± SD; *p< 0.05; Mann-Whitney U; n_*PBS*_ = 6; n_*TLR4Ab*_ = 5).

Highly interestingly, inhibition of the innate immune receptor TLR4 by the use of MTS510 led to an increase of T-cell counts in the ischemic and non-ischemic hemisphere of mice 48h after 45min of MCAO when compared to vehicle (PBS) treated animals (CD3^+^ cells [mean ± SD / 2 x 10^5^ cells] in the ischemic/ipsilateral hemisphere: PBS_ipsi_ 4.8 ± 10.9, TLR4mAb_ipsi_ 95.5 ± 152.0, p 0.0106; CD3^+^ cells in the contralateral hemisphere: PBS_contra_ 0.4 ± 0.5, TLR4mAb_contra_ 29.9 ± 28.3, p 0.0308). (Fig 6A). The determination of B-cell numbers, similar to the determination of activated macrophages/microglia, also revealed an increase of absolute B-cell numbers in the contralateral hemisphere of anti-TLR4-mAb treated mice after MCAO (CD45b.220^+^ cells [mean±SD/2x10^5^cells]: PBS_contra_ 3.3 ± 3.4, TLR4mAb_contra_ 22.0 ± 27.5; p 0,0628). (Fig 6B). Thus, anti-TLR4-treatment alters both the adaptive and innate immune responses after MCAO.

## Discussion

Anti-TLR4 antibody was shown to reduce infarct volumes and neurological deficit at 48h after MCAO in a standard stroke model of transient focal cerebral ischemia similar to the protective effects of anti-TLR2 antibody [8] and intracerebroventricular injection of TLR4-NOX4 inhibitor resatorvid [14] in experimental stroke. The protective effect in our model depends on the route of application (*i*.*a*. > *i*.*v*.; no significant effect after *i*.*p*.). However, direct comparison of the routes of application is hindered by the fact that anti-TLR4 antibody was applied in different amounts, at different time points, and either once (*i*.*a*.) or twice (*i*.*v*. and *i*.*p*.). Recent *in vitro* data using anti-TLR2 antibody OP301 (ref. [16]) and anti-TLR4-antibody 15C1 (ref. [17]), as well as anti-TLR4-antibody E53 *in vivo* (ref. [18]) demonstrated a dose-dependent effect. Thus, MTS510 was applied twice when technically easily practicable (*i*.*v*. and *i*.*p*.) to enable a sufficient concentration in the whole body, as it was performed in similar experiments by other groups previously [26,27]. Moreover, *i*.*p*. antibody application could result in only a low concentration of the antibody in the ischemic tissue due to an insufficient transport to the brain. Dilution effects could also occur if mAb were to be applied systemically (*i*.*v*., or *i*.*p*.) instead of *i*.*a*.. Accordingly, a very recent report showed that also intraperitoneal application of a TLR4-inhibitor (TAK-242) is able to reduce ischemic damage 24h after 60min MCAO [28]. The protective effect of *i*.*a*.-applicated MTS510 at the start of middle cerebral artery occlusion (MCAO) is very robust and highly reproducible, but *i*.*v*. application at the end of MCAO also significantly protects against ischemic brain injury. The lack of significant protection in the long-term evaluation however must be considered within the limits of the high mortality of stroked mice [29] and the fact that those animals which survived the experimental stroke made too rapid a neurological recovery to show significant differences. We can however state that neither an evaluation of surviving animals with regard to neurological score, infarct volumes or neuronal cell count nor a Kaplan-Meier analysis of animal survival revealed any inverse (or detrimental) effect of anti-TLR4 antibody treatment, as was described by Bohacek [9] in TLR2-deficient mice. However, a comparison of these results is potentially complicated by a different dynamic of the infarct development, which occurs with delay if occlusion times are reduced [29].

Flow cytometry analysis of cells involved in the immune response revealed a reduced ratio of macrophage/microglia activation, as was described in TLR2-deficient mice [9]. However, this reduced ratio is mainly based on an increase of activated macrophage/microglia cell count in the contralateral/non-ischemic hemisphere in anti-TLR4-treated animals (Fig 5A and 5B). Interestingly, TLR4-inhibition not only modifies the inflammatory immune response or the degree of brain swelling (Fig 3) at 2d after MCAO, but also alters the adaptive immune response, as measured by infiltrating T-cell and B-cell numbers. Despite an analysis only at 48h after reperfusion, our data argues for a ‘disinhibitory’ effect of a TLR4-blockade, potentially via inhibition of TLR4 mediated regulation of regulatory T-cells [30]. Also indirect effects on cells of the adaptive immune system via TLR4-regulated APCs can be envisioned. Our findings are in contrast to the delayed T-cell recruitment which was observed in TLR-deficient mice in models of axonal degeneration and brain lesion models [4,31]. However, it still remains to be evaluated if perturbation of the ischemic neuroinflammation by TLR4 inhibition on both the innate and adaptive immune response levels also influences the long-term prognosis after stroke. We speculate that timely limited TLR inhibition, as it could be achieved by the use of blocking antibodies could circumvent detrimental effects observed in mice with a constitutive TLR-deficiency [9]. A more profound evaluation (including other species) both of the long-term effects of TLR4 inhibition on the immune system and the long-term outcome of anti-TLR4-mAb treated animals after MCAO is recommended before clinical studies with TLR4-blocking antibodies are initiated.

## Conclusion

Our results demonstrate a robust neuroprotective effect of anti-TLR4-antibody MTS510 after focal cerebral ischemia, if applied intravascularly (*i*.*v*.*; i*.*a*.). Additionally, long-term observation of mice treated with MTS510 does not support the hypothesis of a detrimental effect of antibody treatment. Moreover, our results demonstrate that inhibition of the innate immune receptor TLR4 also alters post-stroke adaptive immune responses in both ischemic and non-ischemic brain tissue. Thus, TLR4 inhibition represents an attractive potential therapeutic option for stroke treatment. However, further experimental studies are required before clinical studies are initiated.

## Supporting Information

S1 Fig Neuronal cell counts and evaluation of inflammatory cells in ischemic and non-ischemic hemisphere after MCAO of mice treated with anti-TLR4 mAb *i*.*p*. (PDF)

S2 Fig Temperature and weight of anti-TLR4-mAb and vehicle-treated mice up to 14d after 15min MCAO. (PDF)

S3 Fig Evaluation of ischemic injury at 14d after 15min MCAO. (PDF)

S4 Fig Stroke volumes and neuronal survival in animals treated with anti-TLR4 antibody *i*.*a*. after 14days. (PDF)

S5 Fig Kaplan-Meier analysis of animal survival of wild-type mice after 15min MCAO treated with or without anti-TLR4 antibody *i*.*a*.. (PDF)

S1 Text Supplemental methods. (PDF)
