# Supplementary material for: Evaluation of the Therapeutic Potential of Anti-TLR4-Antibody MTS510 in Experimental Stroke and Significance of Different Routes of Application
Source: PLoS One. 2016 Feb 5;11(2):e0148428. doi: 10.1371/journal.pone.0148428 (PMC4746129; doi:10.1371/journal.pone.0148428)
Supplement: S1 Fig — (PDF) [file pone.0148428.s001.pdf]

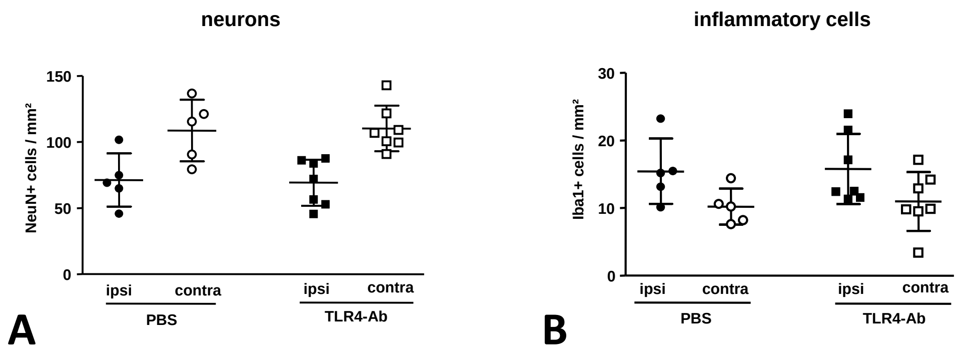

S1 Figure. Neuronal cell counts and evaluation of inflammatory cells in ischemic and non-ischemic hemisphere after MCAO of mice treated with anti-TLR4 mAb *i.p.* MTS510 mAb (**TLR4-Ab**), was applied *i.p.* at the end of MCAO and once again after 24h of reperfusion in a dose of 1µg/animal in each application. Animals treated with injection of vehicle (**PBS**) were used as control. NeuN positive cells were counted in 3 predefined boxes in ipsilateral/ischemic hemisphere (**ipsi**) and contralateral/non-ischemic hemisphere (**contra**) (**Figure IA**). To evaluate if anti-TLR4-treatment affects inflammatory cell accumulation in ischemic brain tissue, Iba1-positive cells (representing macrophages/monocytic cells) were counted at 48h after induction of ischemia in whole ipsilateral (**ipsi**) and contralateral/non-ischemic (**contra**) hemisphere of either vehicle control (PBS), or MTS510-treated (TLR4-Ab) animals (**Figure 1B**). Analysis was performed by investigators blinded to the groups of treatment, values are presented with the mean ± SD (n_{PBS} = 5; n_{TLR4Ab} = 7).
