# Supplementary material for: Evaluation of the Therapeutic Potential of Anti-TLR4-Antibody MTS510 in Experimental Stroke and Significance of Different Routes of Application
Source: PLoS One. 2016 Feb 5;11(2):e0148428. doi: 10.1371/journal.pone.0148428 (PMC4746129; doi:10.1371/journal.pone.0148428)
Supplement: S2 Fig — (PDF) [file pone.0148428.s002.pdf]

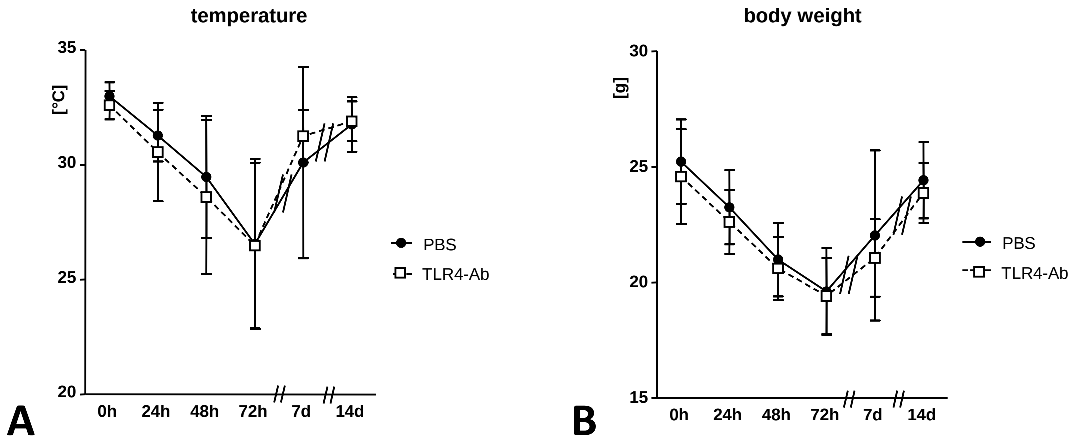

S2 Figure. Temperature and weight of anti-TLR4-mAb and vehicle-treated mice up to 14d after 15min MCAO. Mean temperature (**A**) and mean body weight (**B**) combined with the SD of each value after 15min MCAO from day 0 up to day 14 is shown for both groups, vehicle(PBS)- and anti-TLR4-treated C57Bl/6 male wild-type mice (n_{PBS} = 17; n_{TLR4} = 18).
