# Supplementary material for: Evaluation of the Therapeutic Potential of Anti-TLR4-Antibody MTS510 in Experimental Stroke and Significance of Different Routes of Application
Source: PLoS One. 2016 Feb 5;11(2):e0148428. doi: 10.1371/journal.pone.0148428 (PMC4746129; doi:10.1371/journal.pone.0148428)
Supplement: S3 Fig — (PDF) [file pone.0148428.s003.pdf]

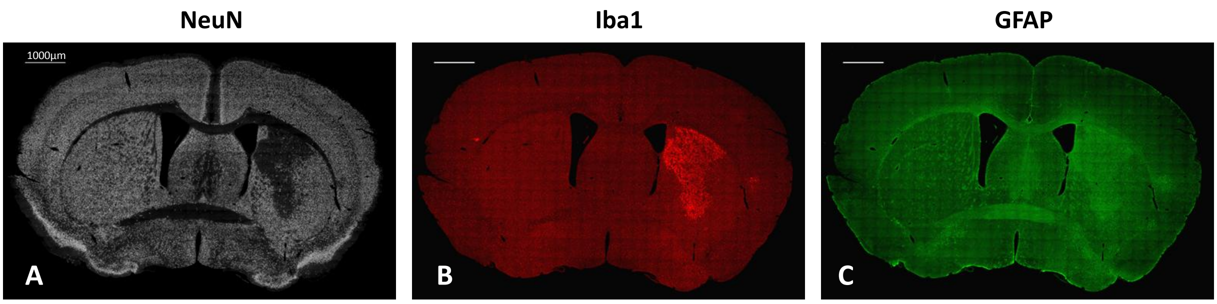


**S3 Figure. Evaluation of ischemic injury at 14d after 15min MCAO.** Sections of a representative mouse brain at 14d after 15min MCAO which were stained with anti-NeuN antibody (neurons) (**A**), anti-Iba1 antibody (macrophages/monocytes and microglia) (**B**), and the astrocytic marker GFAP (**C**) are shown.
