# Supplementary material for: Evaluation of the Therapeutic Potential of Anti-TLR4-Antibody MTS510 in Experimental Stroke and Significance of Different Routes of Application
Source: PLoS One. 2016 Feb 5;11(2):e0148428. doi: 10.1371/journal.pone.0148428 (PMC4746129; doi:10.1371/journal.pone.0148428)
Supplement: S4 Fig — (PDF) [file pone.0148428.s004.pdf]

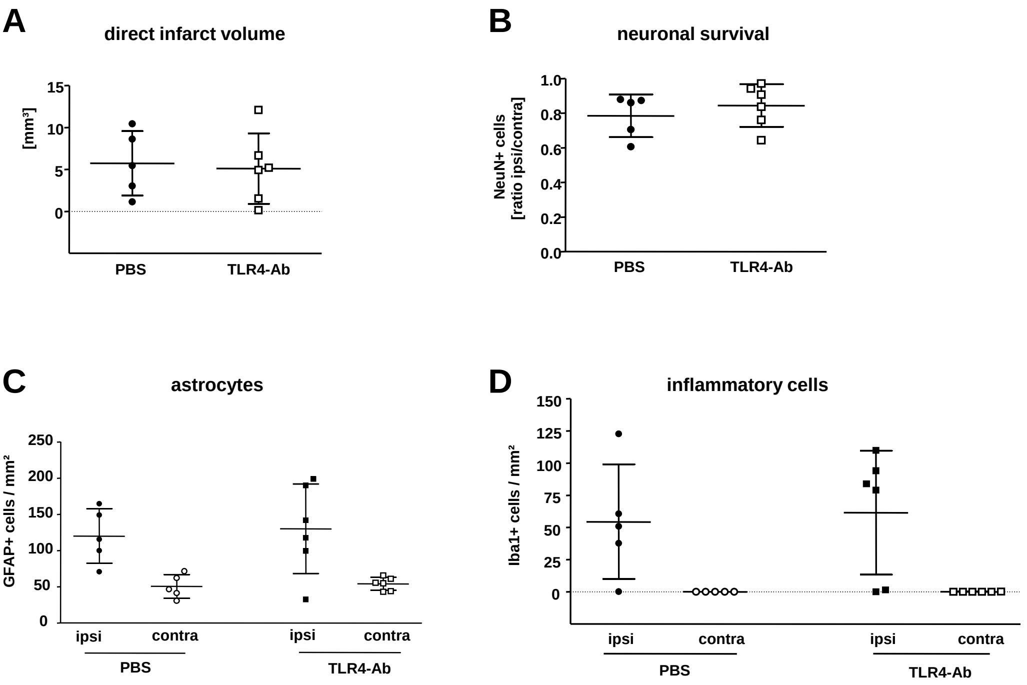

S4 Figure. Stroke volumes and neuronal survival in animals treated with anti-TLR4 antibody *i.a.* after 14days. Infarct volumes were determined after 15min MCAO at day 14 after start of reperfusion by the use of FluoroJade C staining (**A**) after intra-arterial application of anti-TLR4 mAb (**TLR4-Ab**), or vehicle only (**PBS**). The number of surviving neurons was calculated by the ratio of NeuN-positive cell count of ipsilateral/ischemic hemisphere divided by the cell count in the corresponding non-ischemic/contralateral hemisphere (**B**). Activated astrocytes were demonstrated in the ipsilateral/ischemic and contralateral/non-ischemic hemisphere (**C**), as well as inflammatory cells (**D**) by the use of anti-GFAP-specific, and anti-Iba1-specific antibodies, respectively (single values are presented with the mean ± SD) (n_{PBS} = 5; n_{TLR4Ab} = 6).
