# Supplementary material for: Evaluation of the Therapeutic Potential of Anti-TLR4-Antibody MTS510 in Experimental Stroke and Significance of Different Routes of Application
Source: PLoS One. 2016 Feb 5;11(2):e0148428. doi: 10.1371/journal.pone.0148428 (PMC4746129; doi:10.1371/journal.pone.0148428)
Supplement: S5 Fig — (PDF) [file pone.0148428.s005.pdf]

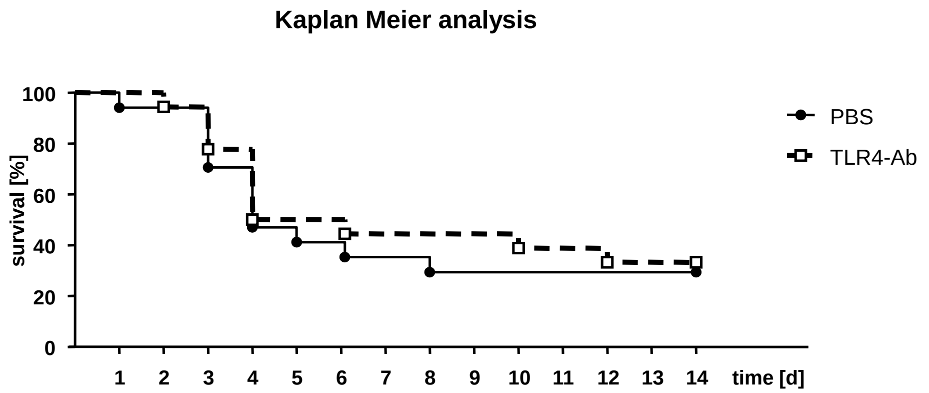

S5 Figure. Kaplan-Meier analysis of animal survival of wild-type mice after 15min MCAO treated with or without anti-TLR4 antibody *i.a.*. The percentage of surviving C57Bl/6N wild-type mice after 15min MCAO with (**TLR4-Ab**) or without (**PBS**) *i.a.* application of anti-TLR4/MD2 mAb from postoperative day 1 up to day 14 after start of reperfusion is shown ($n_{PBS} = 17$; $n_{TLR4} = 18$).
