# Supplementary material for: Evaluation of the Therapeutic Potential of Anti-TLR4-Antibody MTS510 in Experimental Stroke and Significance of Different Routes of Application
Source: PLoS One. 2016 Feb 5;11(2):e0148428. doi: 10.1371/journal.pone.0148428 (PMC4746129; doi:10.1371/journal.pone.0148428)
Supplement: S1 Text — (PDF) [file pone.0148428.s006.pdf]

Supporting information

S1 Text. Supplemental Methods.

Intraaterial application of anti-TLR4 mAb *in vivo*. Intraarterial application was performed before MCAO. The common carotid artery and the external carotid artery were dissected from the surrounding tissue and ligated with 5-0 polyester strings. Next, the internal carotid artery was clipped shortly before the beginning of the middle cerebral artery. A hole was cut into the common carotid artery, a syringe-linked catheter was inserted and placed in front of the microclip and fixed with a 5-0 polyester string. For the time of application the clip was opened, 50 µl of the diluted antibody (1µg/50µl), IgG isotype or PBS, respectively were injected, and closed again to both prevent loss into the extravasal space and ensure its spreading into the future infarct area.

Cerebral ischemia was induced with a 6.0 silicon-coated filament placed into the middle cerebral artery by entering through the common carotid artery and advancing along the internal carotid artery. Brain perfusion was blocked for 45min in the case of 48h of reperfusion and 15min in case of a reperfusion time of 14d. 15min MCAO was applied to reduce the well-known high mortality rate of mice after MCAO beyond 2d of reperfusion, which was also observed in pilot experiments with occlusion times of 30min, 45min, and longer (data not shown).[1] Afterwards the filaments were withdrawn and blood flow was restored. For the time of surgery, a heating pad maintained the body temperature of the animals at 37°C. Intraperitonal and intravenous drug administration were performed directly after the occlusion and 24h later. Efficiency of occlusion and reperfusion of the middle cerebral artery was monitored by Laser Doppler flowmetry (Perimed, Stockholm, Sweden).

Animals with a reduction of initial perfusion of less than 70% were excluded from further evaluation.

Animals that were part of the long-term experiment (n = 40; up to 14d of reperfusion) were provided ‘intensive care’ consisting of application of 100µl ceftriaxone (200mg/kg body weight) intraperitoneally and 500 µl saline solution subcutaneously at the beginning of reperfusion and 24 and 48 hours later to prevent systemic dehydration and to reduce post-stroke mortality.[2,3,4,5] Before and directly after suturation ointment containing dexpanthenole was placed onto the animals eyes to prevent dehydration. Analgetic treatment included intraperitonally applied buprenorphine (0.1 mg/kg body weight) during surgery and lidocaine gel placed onto the sutures directly after suturation as well as 24 hours after MCAO. The animal cages were kept on heating pads to maintain a constant cage temperature of 24 C° until 72h after reperfusion. Even though body weight and surficial body temperature were only documented and analysed before MCAO and 24, 48 and 72 hours as well as 7 and 14 days after reperfusion, the animals were daily seen for health monitoring.

Infarct volumetry. The size of the ischemic lesion 14d after reperfusion was measured with FlouroJade C (Histo-Chem, Inc., Jefferson, USA) in brain slices at interaural positions 0.40mm, 2.34mm, and 4.42mm. Staining was performed according to the recommendations of the manufacturer, and was combined with DAPI (4′,6-Diamidin-2-phenylindol; AppliChem GmbH, Darmstadt, Germany) to counterstain cell nuclei. Direct infarct volumes were calculated by multiplying the means of two slices with their distance (2mm) and summarizing them to a total volume.[6]

Neurological Scoring. Additionally, a modified Neurological Severity Score (mNSS) which consists of three motor tests (exploration of a new cage, climbing and balance) and two sensory tests (body proprioception and pinna reflex) was applied. Each test was evaluated by

a score between 3 and 0, whereas 3 equals no deficit and 0 death. The scores are added up to a score between 0 (death) and 15 (no neurological impairment).[7,8,9]

Cell quantification after 14days of reperfusion. For immunohistochemical-based cell quantification after 14d, brain slices at interaural position 4,42mm (±0,3mm) were selected. Cells were counted using Adobe Photoshop CS4 Extended, Version 11.0 (Adobe Systems, Inc., USA) and AxioVision40 V 4.8.2.0 (Zeiss, Jena, Germany). Iba1-positive cells were counted in the entire brain hemispheres, whereas the counting of NeuN- and GFAP-positive cells, three boxes of 150 ± 15 µm height covering the complete width of the brain section were placed in each brain slide at the following positions: one below the corpus callosum, one at the lower edge of the lateral ventricles, and one at the caudal part of the medial preoptical area. Afterwards, the mean cell number per mm^2 was calculated for each hemisphere.

Supplemental References

1. Baron JC, Macrae IM, Adams HP Jr, Dirnagl U. ESC-BRAIN: experimental and clinical stroke research--do they connect?. Meeting report of the ESC-BRAIN joint symposium held in London and Shanghai in May 2013. *Cerebrovasc Dis.* 2013;36:306-21

2. Prass K, Meisel C, Höflich C, Braun J, Halle E, Wolf T, et al. Stroke-induced immunodeficiency promotes spontaneous bacterial infections and is mediated by sympathetic activation reversal by poststroke T helper cell type 1-like immunostimulation. *J Exp Med.* 2003;198:725-736.

3. Meisel C, Prass K, Braun J, Victorov I, Wolf T, Megow D, et al. Preventive antibacterial treatment improves the general medical and neurological outcome in a mouse model of stroke. *Stroke J Cereb Circ.* 2004;35:2-6.

4. Thöne-Reineke C, Neumann C, Namsolleck P, Schmerbach K, Krikov M, Schefe JH, et al. The beta-lactam antibiotic, ceftriaxone, dramatically improves survival, increases glutamate uptake and induces neurotrophins in stroke. *J Hypertens*. 2008;26:2426-2435.

5. Nederkoorn PJ, Westendorp WF, Hooijenga IJ, de Haan RJ, Dippel DWJ, Vermeij FH, et al. Preventive antibiotics in stroke study: rationale and protocol for a randomised trial. *Int J Stroke Off J Int Stroke Soc.* 2011;6:159-163.

6. Encarnacion A, Horie N, Keren-Gill H, Bliss TM, Steinberg GK, Shamloo M. Long-term behavioral assessment of function in an experimental model for ischemic stroke. *J Neurosci Methods.* 2011;196:247–257.

7. Garcia JH, Wagner S, Liu KF, Hu XJ. Neurological deficit and extent of neuronal necrosis attributable to middle cerebral artery occlusion in rats. Statistical validation. *Stroke J Cereb Circ.* 1995;26:627–634.

8. Hua F, Ma J, Ha T, Kelley JL, Kao RL, Schweitzer JB, Kalbfleisch JH, Williams DL, Li C. Differential Roles of TLR2 and TLR4 in acute focal cerebral ischemia/reperfusion injury in mice. *Brain Res.* 2009;1262:100–108.

1. Li Y, Chopp M, Chen J, Wang L, Gautam SC, Xu YX, Zhang Z. Intrastriatal transplantation of bone marrow nonhematopoietic cells improves functional recovery after stroke in adult mice. *J Cereb Blood Flow Metab.* 2000;20:1311–1319.
